# Subcutaneous Adipose Tissue Measured by B-Mode Ultrasound to Assess and Monitor Obesity and Cardio–Metabolic Risk in Children and Adolescents

**DOI:** 10.3390/biology10050449

**Published:** 2021-05-20

**Authors:** Karin Schmid-Zalaudek, Bianca Brix, Marietta Sengeis, Andreas Jantscher, Alfred Fürhapter-Rieger, Wolfram Müller, Edna N. Matjuda, Muhau M. Mungamba, Benedicta Nkeh-Chungag, Per Morten Fredriksen, Nandu Goswami

**Affiliations:** 1 Otto Loewi Research Center for Vascular Biology, Physiology Division, Immunology and Inflammation, Medical University of Graz, 8010 Graz, Austria; bianca.brix@medunigraz.at (B.B.); an.jantscher@medunigraz.at (A.J.); 2 Gottfried Schatz Research Centre, Biophysics Division, Medical University of Graz, 8010 Graz, Austria; m.sengeis@leistungssport.at (M.S.); alfred.fuerhapter@medunigraz.at (A.F.-R.); wolfram.mueller@rotosport.at (W.M.); 3 Department of Human Biology, Faculty of Health Sciences, Walter Sisulu University, Mthatha 5117, Eastern Cape Province, South Africa; 217297331@wsu.ac.za (E.N.M.); mmungamba@wsu.ac.za (M.M.M.); 4 Department of Biological and Environmental Sciences, Faculty of Natural Sciences, Walter Sisulu University, Mthatha 5117, Eastern Cape Province, South Africa; bnkehchungag@wsu.ac.za; 5 School of Health Sciences, Kristiania University College, Kristiania University, 0107 Oslo, Norway; PerMorten.Fredriksen@kristiania.no

**Keywords:** subcutaneous adipose tissue, ultrasound, childhood overweight/obesity, adolescent overweight/obesity, BMI, cardio–metabolic risk, body fat assessment

## Abstract

**Simple Summary:**

The prevention and treatment of childhood and adolescent overweight and obesity raises the need for accurate body fat assessment. Precise methods are at high technical expense, require exposure to ionizing radiation and are limited to institutional investigations, while common body indicators fail to identify excess body fat. Subcutaneous adipose tissue measured by ultrasound is an alternative approach, which was evaluated in relation to commonly applied body indicators to assess cardio–metabolic risk and its applicability in the field. Subcutaneous adipose tissue measured by ultrasound indicated a clear advantage over commonly applied body indicators and implies that severe body fat assessment errors are to be expected when BMI is used as a measure for body fatness in children. Children or adolescents with identical BMIs may have large differences (>200%) in their amount of subcutaneous adipose tissue. Ultrasound provides an easily applicable, reliable and safe method for accurate assessment of obesity and monitoring treatment responses in children and adolescents at cardio–metabolic risk.

**Abstract:**

Monitoring of children at heightened risk of cardio–metabolic diseases raises the need for accurate assessment of obesity. A standardized approach for measuring subcutaneous adipose tissue (SAT) by bright-mode ultrasound was evaluated in relation to body indices and anthropometry in a cross-sectional sample of 76 South African children (7–10 years) and 86 adolescents (13–17 years) to assess cardio–metabolic risk. SAT was higher in girls as compared to boys (children: 50.0 ± 21.7 mm > 34.42 ± 15.8 mm, adolescents: 140.9 ± 59.4 mm > 79.5 ± 75.6 mm, *p* < 0.001) and up to four times higher in adolescents than in children. In children, measures of relative body weight showed only a poor correlation to SAT (BMI: r = 0.607, *p* < 0.001), while in adolescents, BMI correlated high with SAT (r = 0.906, *p* < 0.001) based on high rates of overweight and obesity (41.8%). Children with identical BMIs may have large differences (>2–3-fold) in their amount of SAT. The moderate association to systolic (r = 0.534, r = 0.550, *p* < 0.001) and diastolic blood pressure (r = 0.402, r = 0.262, *p* < 0.001) further substantiates that SAT measured by ultrasound provides an accurate, safe and easy applicable approach for monitoring in children and adolescents at cardio–metabolic risk.

## 1. Introduction

In the prevention and control of non-communicable diseases (NCDs) as one of the global health challenges [1], childhood and adolescent overweight and obesity is targeted. Along with immediately impaired health and quality of life in affected children, childhood obesity is likely to track into adulthood, causing a heightened risk for and premature onset of a number of serious diseases [2,3,4,5]. Low- and middle-income countries and minority ethnic groups report the most rapid increase in childhood overweight and obesity; about 75% of the 42 million children (<5 years) affected live in Asia and Africa [2,6,7]. Socioeconomic transition and related lifestyle changes such as the quality and quantity of food intake, reduced physical activity and increased screen hours mainly account for accumulative rates of obesity and related health outcomes, especially in adolescents [2,8,9,10].

Body composition and growth rate have a high impact on childhood health and physical performance [11]. In the pediatric routine, percentile curves for growth rate, height and weight for age, together with the body mass index (BMI = m/h^2^) are used as diagnostic criteria, but with limited significance [12,13,14,15,16]. Overweight and obesity are defined by the BMI, applying age- and gender-specific International Obesity Task Force (IOTF) percentile curves of 25 kg/m^2^ for overweight and 30 kg/m^2^ for obesity [17]. The BMI, however, does not consider body composition, as it is not capable of distinguishing between body mass resulting from muscle or from fat. The BMI has a low sensitivity and fails to identify excess body fat in about a quarter up to a half of children with excess body fat [12,13,16,18,19]. In a recent study on pre-school children, the BMI was not only correlated to fat mass (as measured by air displacement plethysmography) but, in the same extent, to fat-free mass, underlining its questionable application as a diagnostic tool for obesity [20]. Problems arise, in particular, when a person’s shape deviates from the norm, as when legs are shorter or longer than expected for their height [21]. Furthermore, BMI cutoff points are developed for Caucasians and do not account for related risks in different ethnic groups [22]. Supplementary anthropometric measures to assess distinct fat deposits such as waist circumference (WC) and waist-to-hip (WHR = w/hip) and waist-to-height ratios (WHtR = w/h) improved diagnostics in large-scale studies [23,24]. However, their added value in predicting adulthood obesity or cardio–metabolic disease in later life on the individual level is controversial [14,25,26,27].

Quantification of the total amount and relative proportions of body tissue compartments is performed at different levels of accuracy and technical expense [11,25,28]. Dual-energy X-ray absorptiometry (DEXA), computed tomography (CT) and magnetic resonance imaging (MRI) are highly precise methods for measuring body composition [23,25], though their technical expense and exposure to ionizing radiation limits their application. Less detrimental, cost-effective and more feasible methods such as bioelectrical impedance and skinfold measures are, however, at the cost of accuracy [29,30].

Ultrasound has been shown to be a highly precise and reliable alternative approach to the more expensive assessment of subcutaneous adipose tissue (SAT) by DEXA, CT or MRI, requiring measurements in institutional settings (e.g., hospitals, universities) [28,31,32,33,34,35]. The uncompressed SAT thickness is measured, and body mass increases due to fat or muscle gain can accurately be distinguished [31,34,36]. Avoiding exposure to radiation further substantiates its application in children, also providing a method for the follow-up of changes in response to treatment [28,29].

Several studies implicate that SAT at the waist is associated with adverse cardio–metabolic risk factors, particularly in children and different ethnicities [37,38,39,40,41,42,43,44,45]. In a large-scale study on Chinese children aged 6–18 years, SAT measured by DEXA was linked to heightened total cholesterol, high low-density and low high-density lipid cholesterol and higher triglycerides [41], implicating its role in the development of cardio–metabolic diseases.

The present study assessed SAT patterning by a recently developed standardized B-mode ultrasound approach in the field. Children and adolescents from a rural South African region with a particularly high prevalence of child and adolescent obesity and hypertension were studied [46,47,48,49]. Anthropometric measures, body indices and hemodynamic parameters were related to SAT patterns to achieve an estimate of their value in discriminating children and adolescents at risk of cardio–metabolic disease. Furthermore, the concordance between SAT patterning based on eight-site measurements compared to four- and five-site measurements was assessed to quantify the efficiency of this time-saving approach [34].

## 2. Materials and Methods

### 2.1. Study Design and Participants

The study was a cross-sectional cohort study conducted between March and August 2019 at selected primary schools Eastern Cape Province, South Africa, involving healthy male and female children (aged 7–10 years) and adolescents (aged 13–17 years). All children/adolescents were of African ancestry. Ethical approval was granted by the Health Sciences Ethics Committee of Walter Sisulu University, South Africa (clearance certificate references numbers: 031/2016 and 014/2014). Written informed consent was obtained from the children’s/adolescents’ parents/legal guardians for voluntary participation. The study was conducted according to the principles stated in the Declaration of Helsinki (2013).

### 2.2. Anthropometry

Anthropometric measurements were performed according to the International Standards for Anthropometric Assessments by local trained fieldworkers [50]. Waist, hip, thigh, calf, ankle and mid-upper arm (MUAC) circumferences were measured (in cm) with an anthropometric tape. Height (h), sitting height (s) and leg lengths (l) were measured to the nearest 0.1 cm by an anthropometer (GPM 100), while body mass (m) was assessed by a wireless calibrated weight scale (Tanita body composition scale). BMI = m/h^2^ (in kg/m^2^), the mass index (MI = 0.53 m/(hs) in kg/m^2^), accounting for the individuals’ leg lengths [30,34], and the Cormic index (C = s/h) [34] were calculated. Age- and gender-specific percentile (pc) curves for BMI (International Obesity Task Force) were applied for classification of normal weight (<85th pc), overweight (≥85th < 95th pc) and obesity (≥95th pc) [17].

### 2.3. Subcutaneous Adipose Tissue (SAT) Measurements

SAT was measured with a portable diagnostic B-mode (brightness mode) ultrasound system (Esaote laptop color Doppler system, SMT, Germany) using a linear probe operated at 18 MHz (harmonic mode, axial resolution: 0.10–0.l5 mm). Measures were performed by experienced sports scientists from the same laboratory. Marking of the eight standardized sites was done in standing and sitting positions [31,34], while for image capturing, participants were lying in supine, prone, or rotated position. To avoid compression, the ultrasound probe was placed above each site without pressure, applying a thick layer of ultrasound gel (3–5 mm). Ultrasound images were collected from upper abdomen (UA), lower abdomen (LA), front thigh (FT), lateral thigh (LT), medial calf (MC), c (ES), distal triceps (DT) and brachioradialis (BR) [31,34]. An interactive image segmentation algorithm was applied for detection of the SAT contour, and the software (NISOS–BCA–F 4.0; rotosport.at) automatically performed a series of thickness measurements of SAT with an accuracy of 0.1–0.2 mm [32,33,34]. Reliability studies in various groups have shown that the limit of agreement (LOA) for the sums of the eight thickness measurements is between 1 and 2 mm [30,31,33,34]. Sound speed for distance determination in SAT was set to c = 1450 ms^−1^. Each image revealed multiple (between 50 and 300, depending on the breadth of the region of interest) thickness measurements of a selected site. Mean, standard deviation (SD), median, minimum and maximum are calculated automatically. The mean SAT thickness at a given site (d) is computed either with fibrous structures included (d_i_) or excluded (d_e_), providing also quantification of the number of fibrous structures (fasciae) embedded in the SAT (df = d_i_ − d_e_). D_I_ represents the sum of SAT thicknesses of all eight sites with fibrous structures included, and D_E_ with fibrous structures excluded, while F and F% represent fibers in mm and in percent, respectively. Additionally, the estimated SAT mass in kg and the percentage of SAT mass with respect to body mass are calculated. For assessments in larger samples, calculation of the parameters based on only 4- or 5-site measurements is provided, allowing a quicker application at a marginal loss of accuracy and reliability [34].

### 2.4. Blood Pressure Measurements

Resting blood pressure was measured three times after a five-minute rest in seated position, automatically at 2 min intervals, in a quiet room using the Omron automated sphygmomanometer (HBP-1100; Omron Healthcare Co., Ltd., Lonato Del Garda, Italy). The average of three blood pressure readings was used for analyses.

### 2.5. Statistical Analysis

A priori sample size calculation for differences between girls and boys considering a large effect (d_Hedges_ = 0.517) as reported in children [29], α = 0.05, power = 0.95, revealed a total size of *n* = 52 for comparison of the total SAT and of *n* = 89 for comparisons of the eight standardized sites in a between-groups design analyzed by MANOVA. Normal distribution was examined by Shapiro–Wilks test. Descriptive statistics are presented as mean ± standard deviation (SD). Differences between groups (by sex or BMI) on metric parameters were assessed by Student’s *t*-test, ANOVA or MANOVA at a significance level of α = 0.05. Pearson’s correlations were calculated to determine the relationship between anthropometric measures, SAT and blood pressure. All data were analyzed by SPSS (Version 25.0, IBM Corp., Armonk, NY, USA).

## 3. Results

A total of 76 children (53 girls) with a mean age of 9.13 ± 0.84 years (CI: 8.94, 9.32; range 7–10 years) and 86 adolescents (60 girls) with a mean age of 14.94 ± 0.97 years (CI: 14.73, 15.15; range: 13–17 years) were included. Three children (2 girls, 1 boy) and one adolescent boy had extremely high BMIs (35.6; 39.4; 31.9; 45.8) and were excluded from descriptive statistics to obtain sample appropriate mean values (for details of excluded cases please refer to Table S1 in the Supplementary Materials).

### 3.1. Sexual Dimorphism in Anthropometric Measures and Subcutaneous Adipose Tissue

In children neither BMI, MI nor the Cormic index differed significantly between girls and boys (Table 1). MANOVA of anthropometric measures (including the eight circumferences listed in Table 1) revealed an overall effect for sex (F (8, 62) = 2.097, *p* = 0.049, Wilks-λ = 0.79, η^2^ = 0.21) based on larger mid-upper arm (MUA) and thigh circumferences in girls as compared to boys (means ± SDs, test statistics see Table 1). MANOVA of the SAT measures indicated a significant overall effect for sex (F (8, 64) = 3.74, *p* = 0.001, Wilks-λ = 0.68, η^2^ = 0.32), based on larger amounts of SAT in girls as compared to boys at each of the eight sites (Table 1). Correspondingly, the sum of SAT of all eight sites also differed significantly between girls and boys. The amount of SAT including fibers (D_I_) was 19.6 mm (36.3%) and SAT excluding fibers (D_E_) was 19.1 mm (38.6%) larger in girls as compared to boys. Fibrous structures did not differ significantly, while SAT in kg and % did (see Table 1).

In adolescents, only the BMI indicated significant differences between male and female adolescents, but not the MI or Cormic index (Table 2). Anthropometric measures showed a significant interaction with sex (F (8, 73) = 11.57, *p* < 0.001, Wilks-λ = 0.44, η^2^ = 0.56) based on larger neck circumferences in male (*p* = 0.007) and larger hip and chest circumferences in female (*p* = 0.015) adolescents. MANOVA of SAT measures indicated a dependency on sex (F (8, 74) = 4.204, *p* < 0.001, Wilks-λ = 0.69, η^2^ = 0.31) based on larger amounts of SAT in female as compared to male adolescents at each of the eight sites (Table 2). The total sum of SAT at all eight sites including fibers (D_I_) was 61.4 mm (43.6%) and SAT excluding fibers (D_E_) 61.5 mm (45.8%) larger in adolescent girls as compared to boys. Fibrous structures did not differ significantly, while SAT in kg (t (83) = 3.317, *p* < 0.001) and % (t (82) = 4.593, *p* < 0.001) did (see Table 2).

### 3.2. SAT in Children and Adolescents

Comparison between children and adolescents indicated significantly larger amounts of SAT at each of the eight sites (F (8, 148) = 16.85, *p* < 0.001, Wilks-λ = 0.52, η^2^ = 0.48), as well as of the total amount of SAT in mm (D_I_: t (104) = −10.16, *p* < 0.001 and D_E_: t (103) = −10.07, *p* < 0.001), kg (t (98) = −12.01, *p* < 0.001) and percent (t (142) = −7.62, *p* < 0.001) in adolescents. SAT in adolescents was particularly larger at the upper (18.74 ± 14.3 mm vs. 4.29 ± 3.73 mm) and lower (26.35 ± 17.1 mm vs. 7.70 ± 4.87 mm) abdomen as compared to children (see Figure S1 in the Supplementary Materials). Sex split analyses similarly revealed significantly more SAT in female adolescents as compared to younger girls at each of the eight sites (F (8, 107) = 18.05, *p* < 0.001, Wilks-λ = 0.43, η^2^ = 0.57), with the largest difference at the upper abdomen (21.07 ± 13.68 mm vs. 5.03 ± 4.03 mm), lower abdomen (29.38 ± 15.66 mm vs. 8.67 ± 5.07 mm) and erector spinae (13.19 ± 7.36 mm vs. 3.81 ± 2.72 mm), but also the lateral thigh (32.67 ± 12.84 mm vs. 13.49 ± 4.94 mm). In male adolescents, SAT was also significantly larger as compared to younger boys (F (8, 31) = 3.11, *p* = 0.011, Wilks-λ = 0.55, η^2^ = 0.45), particularly at the erector spinae (5.84 ± 6.13 mm vs. 1.86 ± 1.33 mm), the upper abdomen (9.69 ± 13.22 mm vs. 2.83 ± 2.7 mm) and lower abdomen (14.58 ± 17.72 mm vs. 5.67 ± 3.72 mm, see Figure S1 in the Supplementary Materials).

### 3.3. Correlation of Anthropometric Measures, Body Indices and SAT

Anthropometric measures correlated moderately with corresponding SAT measures of the eight standardized sites with exception of ankle circumference in the child cohort, which is not covered by SAT measures. In children, moderate correlations were found for upper and lower abdomen with waist circumference (r = 0.60, r = 0.66, *p* < 0.001, respectively) and the total amount of SAT and thigh (r = 0.75, *p* < 0.001). In adolescents, high correlations were found for upper and lower abdomen with waist circumference (r = 0.85, r = 0.89, *p* < 0.001, respectively) and hip circumference (r = 0.82, r = 0.86, *p* < 0.001, respectively), as well as for the total sum of SAT (r = 0.90, *p* < 0.001, for details on each of the sites please refer to Table S2 in the Supplementary Materials).

**Figure 1:**
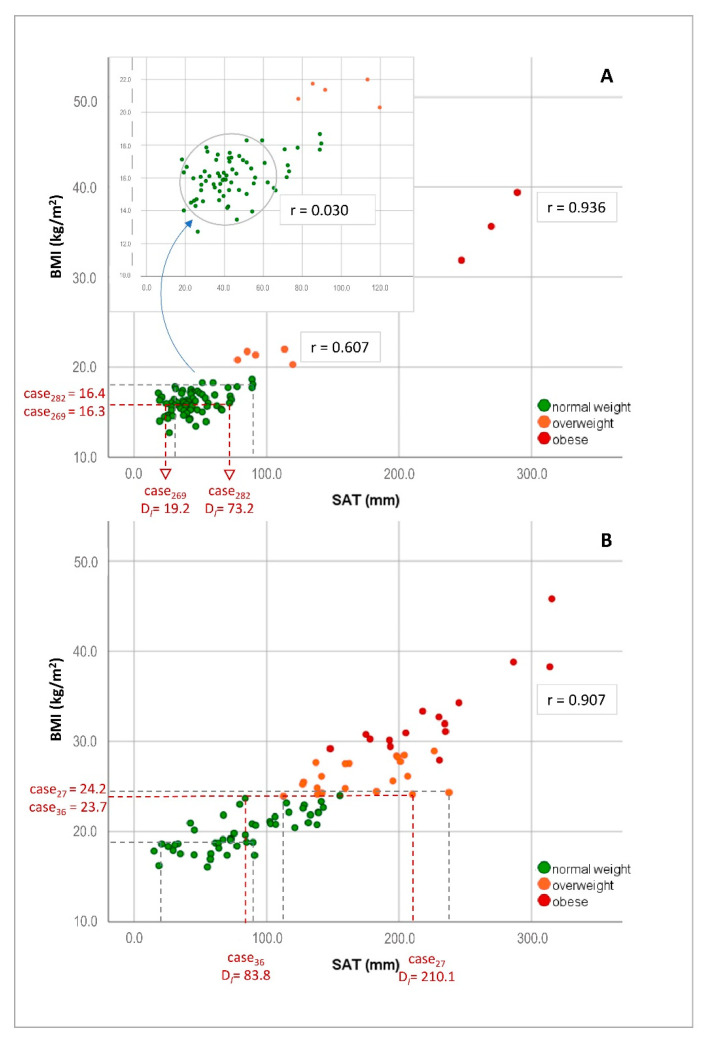
Correlation between subcutaneous adipose tissue (SAT) and BMI in children (**A**) and adolescents (**B**). Note that the correlation in children is biased when including extreme cases and drops almost to zero (r = 0.030) when including only the mean 50% of the child sample. As indicated by two cases in the plot, two children or adolescents with almost identical BMIs may differ largely in their amount of SAT (>200%). Despite the high correlation in adolescents, this holds true for many cases.

In children, the BMI correlated highly with the MI (r = 0.840, *p* < 0.001), but not with the Cormic index (r = 0.156, *p* = 0.186) and only moderately with both the D_I_ (r = 0.670, *p* < 0.001) and D_E_ (r = 0.666, *p* < 0.001). Similarly, the MI correlated moderately to D_I_ (r = 0.565, *p* < 0.001) and D_E_ (r = 0.566, *p* < 0.001), while the Cormic index was not related to SAT measures (r = 0.032, r = 0.034, resp). Correlating the BMI and the total amount of SAT (D_I_) only for the mean part of the sample, represented by the interquartile range (IQR), revealed even almost zero correlation (r_IQR_ = 0.030, *p* = 0.859). Figure 1A displays the correlation between BMI and D_I_ in children. As indicated by the example of two cases (case 269, case 282), children with corresponding BMIs show a large difference in their amount of SAT. The plot shows that this holds true for many other similar cases. In children, WHtR was also only moderately correlated to the total SAT (D_I_: r = 0.407, *p* < 0.001 and D_E_: r = 0.407, *p* < 0.001) and SAT at the upper (r = 0.51, *p* < 0.001) and lower abdomen (r = 0.50, *p* < 0.001, for details please refer to Table S2 in the Supplementary Materials).

**Figure 2:**
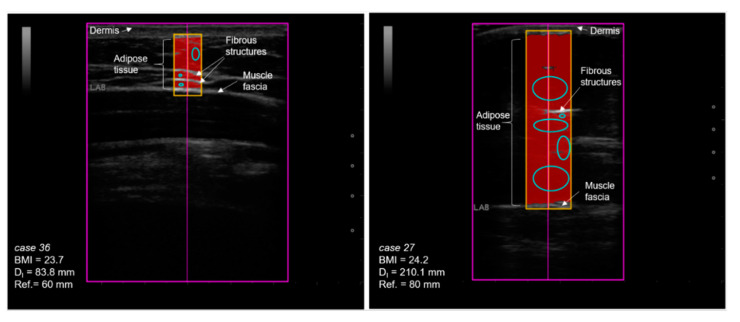
Subcutaneous adipose tissue measured at lower abdomen in two 16-year-old girls with similar BMIs. Note that, although the two girls have almost identical BMIs, the amount of SAT is more than the two-fold in the girl at the right side. Mind that the reference lengths were 6 cm in case 36 (**left**) and 8 cm in case 27 (**right**).

In adolescents, high correlations were found between BMI and MI (r = 0.972, *p* < 0.001) and BMI and D_I_ (r = 0.906, *p* < 0.001) and D_E_ (r = 0.905, *p* < 0.001). Similarly, the MI also showed a good correlation with D_I_ (r = 0.881, *p* < 0.001) and D_E_ (r = 0.879, *p* < 0.001), even when excluding one boy with extreme bodily measures. The Cormic index was neither correlated to the BMI, MI nor any SAT measure. WHtR was also highly correlated to D_I_ (r = 0.856, *p* < 0.001) and D_E_ (r = 0.854, *p* < 0.001) and to the upper (r = 0.80, *p* < 0.001) and lower abdomen (r = 0.82, *p* < 0.001, see also Table S2 in the Supplementary Materials). Figure 1B displays the correlation between BMI and SAT (D_I_) in adolescents. Although the correlation is high, adolescents with identical BMIs (e.g., case 27, female, 16 years and case 36, female, 16 years) but large-scale differences in SAT can be found. Figure 2 further highlights this problem and shows that, although the BMIs of the two same-aged girls are quite similar, a large difference in the amount of subcutaneous fat at the lower abdomen is observed.

### 3.4. Hemodynamic Measures and Subcutaneous Adipose Tissue

For analyses of hemodynamic parameters, highly overweight/obese children and adolescents were included to investigate the effect on blood pressure. In the child sample, moderate correlations between SBP and D_I_ (r = 0.550, *p* < 0.001), D_E_ (r = 0.548, *p* < 0.001) and SAT in kg (r = 0.620, *p* < 0.001) and % (r = 0.425, *p* < 0.001) were observed. In addition, DBP was moderately correlated, while there was no association to HR (see Table S3, upper part in the Supplementary Materials). In adolescents, SBP was also moderately correlated with D_I_ (r = 0.534, *p* < 0.001), D_E_ (r = 0.525, *p* < 0.001) and SAT in kg (r = 0.555, *p* < 0.001) and % (r = 0.436, *p* < 0.001), while DBP showed only low correlations. No association was found between measures of SAT and HR (see Table S3, lower part in the Supplementary Materials).

BMI classification according to the age- and gender-specific percentile curves indicated eight children (10.53%), including the three children with extreme BMIs, as exceeding the 85th percentile. To account for the large sample size difference and related heterogeneous variances, the corrected t-statistic was applied to evaluate group differences between children with normal weight and overweight/obesity (subsumed to one group). Furthermore, non-parametric statistic was applied to confirm the results. Significant differences between lean and overweight/obese children were observed with regard to SBP and D_I_ and D_E_ fibers, as well as SAT in kg and % (Table 3). Children with overweight/obesity had higher blood pressure (SBP: 136 ± 25.6 mmHg > 111 ± 13.0 mmHg; DBP: 83 ± 17.1 mmHg > 69 ± 8.3 mmHg) and a higher amount of SAT (D_I_: 161.8 ± 90.3 mm > 44.2 ± 17.3 mm) as compared to children with normal weight, respectively.

In adolescents, 24.4% (28.8% of the girls, 10% of the boys) were classified as overweight and 17.4% (16.7% of the girls, 20% of the boys) as having obesity (Table 3, lower part). Significant differences between the three groups were found in SBP (F (2, 83) = 14.04, *p* < 0.001, η^2^ = 0.25) but not in DBP and HR. SBP was higher in overweight (126 ± 11.1 mmHg, *p* < 0.001) and obese (124 ± 14.1 mmHg, *p* < 0.001) compared to normal weight (113 ± 8.2 mmHg) adolescents. Furthermore, D_I_ (F (2, 83) = 91.40, *p* < 0.001, η^2^ = 0.69), D_E_ (F (2, 83) = 89.51, *p* < 0.001, η^2^ = 0.68) and fibers (F (2, 83) = 16.35, *p* < 0.001, η^2^ = 0.28), as well as SAT in kg (F (2, 83) = 96.30, *p* < 0.001, η^2^ = 0.70) and % (F (2, 83) = 45.10, *p* < 0.001, η^2^ = 0.52) were significantly higher in overweight and obese adolescents (Table 4).

### 3.5. Estimation of SAT by Four- and Five-Site Measurements

Comparison between the measured SAT (DI, DE, fibers) at the eight standardized sites and the computed estimates of SAT in kg and % based on only four- or five-site measurements (including the first four or five of the eight sites, respectively) indicated high inter-correlations and reliability scores. SAT in kg and % based on eight sites was highly correlated with four-site (D_I4_: r4kg = 0.993, *p* < 0.001, r_4%_ = 0.981, *p* < 0.001, respectively) and five-site measurements (D_I5_: rD_5kg_ = 0.995, *p* < 0.001, rD_5%_ = 0.987 *p* < 0.001, respectively) in the whole cohort (for details please refer to Table S4 in the Supplementary Materials).

## 4. Discussion

The present study on children (7–10 years) and adolescents (13–17 years) evaluated a standardized ultrasound approach for the assessment of subcutaneous adipose tissue (SAT) in the field and its relation to anthropometric measures, body status indicators and association to cardio–metabolic risk. In a sex-specific comparison, fat patterning by ultrasound indicated a clear advantage over commonly applied body indicators (BMI, MI, Cormic index, WHtR), even when based on only four or five site measures. In both groups (children and adolescents), females had larger amounts of SAT, with largest sex difference at the lateral thigh, a fat deposit typical for the physical characteristics of the adult female shape [25,36]. Adolescents of both sexes showed remarkably higher amounts of SAT, about the four-fold at the upper and the three-fold at the lower abdomen compared to children. This corresponds with the high rates of overweight/obesity in adolescents of this area, as 41.8% of the youth were classified as being overweight or obese when applying age- and sex-specific BMI (IOTF) cutoff scores [17] and confirms previous findings [6,46,47,48]. However, in accordance with current literature [29,31,36,42], the BMI was only moderately correlated with SAT in children, underlining that substantial discrepancies have to be faced when comparing BMI (or MI) at the level of individuals. Even in adolescents, where the high correlation between BMI and total SAT is actually based on the accumulation of fat, two adolescents with almost identical BMIs may have large scale differences in SAT (Figure 2), and this holds for many other cases.

Youth with excess adiposity, as determined by DXA, were shown to have significantly higher levels of cardio–metabolic risk factors, independent of the BMI cutoff applied [51]. In particular, in children under 10 years of age, BMI is only a poor predictor of body fat [15,20], underlining the need for more accurate measures of adiposity. Additionally, waist circumference and WHtR did not differ between girls and boys of either group, while SAT at the upper and lower abdomen were considerably higher in girls of both groups.

Hypertension is the most important risk factor for cardio-vascular disease (CVD) and a frequent symptom in overweight/obesity. In parallel to a larger amount of SAT, higher SBP and DBP were observed in children and adolescents with overweight and obesity. We found significant correlations between systolic and, to a lower amount, also diastolic blood pressure and SAT of all sites. Notably, these associations were already observed in children. Multi-detector computed tomography (MDCT) in children showed that systolic blood pressure is associated with higher upper body subcutaneous fat volumes [37,40,42], which is confirmed by our findings. In children and adolescents, subcutaneous adiposity at the waist was shown to even be a better predictor of metabolic syndrome markers than visceral fat [37,41,42,43], further supporting that SAT measured by ultrasound is a promising method for monitoring children at heightened risk of cardio–metabolic disease [28].

The dramatic increase of SAT in adolescents of both sexes, particularly around waist, displays a harmful development. Environmental conditions and lifestyle-related health risk factors, with unhealthy diet, physical inactivity and alcohol and tobacco use as the key sources of increasing obesity [8,52], even in rural South Africa, have to be targeted in the prevention of children and the treatment of adolescents, with the latter being more prone to these risk factors [52,53,54]. SAT measured by ultrasound provides an accurate and safe method not only for the assessment of adiposity but also for following up obesity treatment, as measures can be repeated without hesitation [28].

The present study has several limitations. Firstly, the cross-sectional character of the study does not allow the conclusion of increasing of SAT in adolescence, as this would require longitudinal measures, which were not performed. Additionally, the assessment of Tanner stages for classification of sexual maturation might have further elucidated the present results. Secondly, systolic blood pressure and waist circumference are only two out of five cardio–metabolic syndrome markers, while plasma lipid and blood glucose profiles were not assessed in the given groups of children and adolescents. However, the relation between SAT and these cardio–metabolic risk factors has been repeatedly reported [37,41,42,43]. Thirdly, the different sample size of girls and boys might have biased the present findings. However, we applied robust statistical methods, and the effects observed were large. Finally, we did not compare SAT measurements with other methods to quantify body fat (CT, MRI), as this has previously been shown as outlined above [28].

## 5. Conclusions

Measurement of body composition in children is a challenge, as changes in height, weight and body compartments continue throughout the life span. Severe body fat assessment errors are to be expected when BMI is used as a measure for body fatness in children, questioning its application as a diagnostic tool in pediatrics. Cardio–metabolic complications not only depend on the amount but, substantially, on the distribution of fat. With the ready availability of small and portable devices, subcutaneous fat patterning by ultrasound provides an accurate, easily applicable and safe method for the assessment of fat distribution in children and in the field. Development of age- and sex-specific reference values for SAT as well as changes in SAT and related cardio–metabolic risks in longitudinal studies should be carried out in future studies.

## Figures and Tables

**Table 1:** Anthropometric measures and subcutaneous adipose tissue (SAT) at eight sites in children aged 7–10 years, split by sex.

	Girls (*n* = 50)	Boys (*n* = 23)	Total (*n* = 73)		
	mean ± SD	mean ± SD	mean ± SD (CI)	t _(df)_	* p *
Age	9.64 ± 0.94	9.78 ± 0.90	9.69 ± 0.93 (9.47, 9.01)	−0.609 (71)	0.554
*Anthropometric measures*					
body height (m)	1.30 ± 0.07	1.31 ± 0.07	1.31 ± 0.07 (1.29, 1.33)	−0.704 (71)	0.483
body weight (kg)	28.4 ± 5.1	28.2 ± 4.8	28.3 ± 5.02 (27.2, 29.5)	0.181 (71)	0.857
sitting height (m)	0.683 ± 0.03	0.683 ± 0.03	0.683 ± 0.03 (0.675, 0.690)	−0.022 (71)	0.983
leg length (m)	0.726 ± 0.05	0.739 ± 0.04	0.729 ± 0.05 (0.719, 0.741)	−0.872 (71)	0.386
				F _(1)_	*p*
neck circumference (cm)	26.4 ± 1.79	26.2 ± 1.75	26.3 ± 1.77 (25.9, 26.7)	0.087	0.769
chest circumference (cm)	61.5 ± 4.38	60.4 ± 3.34	61.2 ± 4.11 (60.2, 62.2)	1.073	0.304
mid-upper arm circ. (cm)	20.7 ± 2.37	19.2 ± 2.52	20.3 ± 2.50 (19.7, 20.9)	5.739	0.019
waist circumference (cm)	58.1 ± 5.42	56.4 ± 3.49	57.6 ± 4.96 (56.4, 58.8)	1.676	0.200
hip circumference (cm)	68.8 ± 5.07	66.3 ± 5.29	68.1 ± 5.23 (66.9, 69.3)	3.517	0.065
thigh circumference (cm)	36.7 ± 3.33	33.9 ± 3.88	35.9 ± 3.71 (35.0, 36.7)	9.899	0.002
calf circumference (cm)	26.3 ± 2.72	25.5 ± 2.26	26.1 ± 2.60 (25.5, 26.7)	1.721	0.194
ankle circumference (cm)	20.5 ± 1.53	20.7 ± 1.90	20.5 ± 1.64 (20.2, 20.9)	0.301	0.585
*Body indices*				t _(df)_	*p*
BMI (kg/m^2^)	16.55 ± 1.84	16.14 ± 1.71	16.42 ± 1.79 (16.0, 16.8)	0.905 (71)	0.369
MI (0.53 m/(hs))	17.05 ± 1.95	16.88 ± 2.56	16.99 ± 2.15 (16.5, 17.5)	0.313 (71)	0.755
Cormic index	0.523 ± 0.016	0.518 ± 0.009	0.522 ± 0.014 (.519, 0.525)	1.425 (71)	0.158
WHt ratio (m)	0.423 ± 0.034	0.427 ± 0.024	0.424 ± 0.031 (.417, 0.431)	−0.582 (71)	0.563
*SAT measures (in mm)*				F _(1, 73)_	*p*
upper abdomen (UA)	5.03 ± 4.03	2.83 ± 2.57	4.29 ± 3.73 (3.40, 5.18)	5.782	0.019
lower abdomen (LA)	8.67 ± 5.07	5.67 ± 3.72	7.70 ± 4.87 (6.54, 8.64)	6.441	0.013
front thigh (FT)	7.29 ± 2.21	5.21 ± 1.90	6.64 ± 2.30 (6.09, 7.19)	15.269	<0.001
lateral thigh (LT)	13.49 ± 4.94	7.71 ± 3.63	11.62 ± 5.28 (10.36, 12.88)	25.180	<0.001
medial calf (MC)	5.62 ± 2.22	3.72 ± 1.56	5.01 ± 2.19 (4.49, 5.54)	13.726	<0.001
erector spinae (ES)	3.81 ± 2.72	1.86 ± 1.33	3.13 ± 2.43 (2.55, 3.71)	10.565	0.002
distal triceps (DT)	6.87 ± 2.12	5.27 ± 1.88	6.36 ± 2.16 (5.85, 6.88)	9.624	0.003
brachioradialis (BR)	3.23 ± 1.38	2.15 ± 1.00	2.89 ± 1.37 (2.57, 3.22)	11.338	0.001
				t _(df)_	*p*
D_I_	54.02 ± 21.71	34.42 ± 15.84	47.65 ± 21.7 (42.5, 52.8)	3.875 (71)	<0.001
D_E_	49.54 ± 20.90	30.46 ± 15.13	43.30 ± 20.8 (38.3, 48.3)	3.924 (71)	<0.001
Fibers	4.48 ± 1.53	3.96 ± 1.36	4.34 ± 1.5 (3.9, 4.7)	1.393 (71)	0.168
SAT (kg)	4.15 ± 1.56	2.96 ± 1.33	3.79 ± 1.59 (3.41, 4.17)	3.045 (71)	0.003
SAT (%)	14.44 ± 3.55	10.36 ± 2.89	13.21 ± 3.84 (12.3, 14.1)	4.646 (71)	<0.001

Abbreviations: BMI: body mass index; MI: mass index; WHt: waist to height ratio; SAT: subcutaneous adipose tissue; D_I_: sum SAT including fibers; D_E_: sum SAT excluding fibers, SAT in kg and in %.

**Table 2:** Anthropometric measures and subcutaneous adipose tissue (SAT) at eight sites in adolescents aged 13–17 years, split by sex.

	Girls (*n* = 66)	Boys (*n* = 19)	Total (*n* = 85)		
	mean ± SD	mean ± SD	mean ± SD (CI)	t _(df)_	*p*
Age	15.05 ± 0.92	14.58 ± 1.12	14.89 ± 0.97 (14.67, 15.10)	1.854 (83)	0.067
*Anthropometric measures*					
body height (m)	1.57 ± 0.05	1.60 ± 0.09	1.58 ± 0.06 (1.56, 1.59)	−2.012 (83)	0.048
body weight (kg)	59.7 ± 13.1	55.6 ± 17.4	58.6 ± 14.0 (55.5, 61.7)	1.111 (83)	0.270
sitting height (m)	0.800 ± 0.04	0.801 ± 0.05	0.80 ± 0.04 (.792, 0.809)	−0.088 (83)	0.930
leg length (m)	0.022 ± 0.05	0.912 ± 0.08	0.920 ± 0.05 (.908, 0.932)	0.476 (83)	0.4766
				F _(1, 80)_	*p*
neck circumference (cm)	31.0 ± 1.81	32.2 ± 3.08	31.3 ± 2.22 (30.8, 31.8)	4.776	0.032
chest circumference (cm)	85.5 ± 9.59	79.3 ± 12.64	84.1 ± 10.6 (81.7, 86.4)	5.131	0.026
mid-upper arm circ. (cm)	26.6 ± 3.97	25.4 ± 4.97	26.3 ± 4.22 (25.4, 27.3)	1.138	0.289
waist circumference (cm)	73.2 ± 9.52	73.4 ± 16.01	73.2 ± 11.3 (70.7, 75.7)	0.008	0.928
hip circumference (cm)	100.7 ± 9.99	91.0 ± 14.77	98.5 ± 11.9 (95.8, 101.1)	10.866	0.001
thigh circumference (cm)	52.2 ± 6.92	51.1 ± 10.53	52.0 ± 7.84 (50.2, 53.7)	0.319	0.574
calf circumference (cm)	35.6 ± 4.05	33.9 ± 5.05	35.2 ± 4.34 (34.3, 36.2)	2.515	0.117
ankle circumference (cm)	23.3 ± 2.17	23.2 ± 2.63	23.3 ± 2.27 (22.8, 23.8)	0.055	0.815
*Body indices*				t _(df)_	*p*
BMI (kg/m^2^)	24.11 ± 4.98	21.38 ± 5.12	23.41 ± 5.08 (22.3, 24.5)	2.096 (83)	0.039
MI (0.53 m/(hs))	25.04 ± 5.13	22.64 ± 5.13	24.49 ± 5.19 (23.4, 25.6)	1.795 (83)	0.076
Cormic index	0.510 ± 0.023	0.500 ± 0.013	0.507 ± 0.021 (0.503, 0.512)	1.840 (83)	0.069
WHt ratio (m)	0.469 ± 0.067	0.458 ± 0.092	0.466 ± 0.073 (0.451, 0.482)	0.602 (83)	0.549
*SAT measures (in mm)*				F _(1, 73)_	*p*
upper abdomen (UA)	21.07 ± 13.68	9.69 ± 13.22	18.74 ± 14.3 (15.6, 21.9)	9.483	0.003
lower abdomen (LA)	29.38 ± 15.66	14.58 ± 17.72	26.35 ± 17.1 (22.6, 30.1)	11.433	0.001
front thigh (FT)	15.43 ± 5.52	8.41 ± 6.60	13.99 ± 6.39 (12.6, 15.4)	20.134	<0.001
lateral thigh (LT)	32.67 ± 12.84	15.97 ± 14.40	29.25 ± 14.7 (26.0, 32.5)	21.734	<0.001
medial calf (MC)	11.74 ± 4.58	5.96 ± 3.55	10.55 ± 4.96 (9.5, 11.6)	23.364	<0.001
erector spinae (ES)	13.19 ± 7.36	5.84 ± 3.55	11.68 ± 7.69 (10.0, 13.4)	14.338	<0.001
distal triceps (DT)	11.08 ± 4.57	6.43 ± 5.70	10.13 ± 5.14 (9.0, 11.3)	12.608	0.001
brachioradialis (BR)	6.32 ± 2.48	3.26 ± 2.51	5.69 ± 2.76 (5.1, 6.3)	20.363	<0.001
				t _(df)_	*p*
D_I_	140.86 ± 59.4	79.48 ± 75.6	127.14 ± 67.2 (112.5, 141.8)	3.727 (83)	<0.001
D_E_	134.19 ± 58.5	72.73 ± 72.4	119.84 ± 66.1 (105.4, 134.3)	3.821 (83)	<0.001
Fibers	6.66 ± 2.3	6.75 ± 3.6	6.54 ± 2.4 (6.0, 7.1)	−0.135 (83)	0.893
SAT (kg)	13.13 ± 5.31	8.17 ± 7.06	11.89 ± 5.86 (10.6, 13.2)	3.317 (83)	0.001
SAT (%)	21.25 ± 4.50	13.16 ± 739	19.43 ± 6.25 (18.1, 20.8)	5.910 (83)	<0.001

Abbreviations: BMI: body mass index; MI: mass index; WHt: waist to height ratio; SAT: subcutaneous adipose tissue; D_I_: sum SAT including fibers; D_E_: sum SAT excluding fibers, SAT in kg and in %.

**Table 3:** Hemodynamic measures and subcutaneous adipose tissue (SAT) in children (7–10 years) with normal weight, overweight or obesity.

BMI Children	<85th pc	≥85th < 95th pc	≥95th	Total	
Girls	46 (88.5%)	4 (7.7%)	2 (3.8%)	52 (100%)	
Boys	22 (91.7%)	1 (4.2%)	1 (4.2%)	24 (100%)	
Total children	68 (89.5%)	5 (6.6%)	3 (3.9%)	76 (100%)	
	mean ± SD	mean ± SD		t _(df)_	*p*
SBP	111 ± 13.0	136 ± 25.6		−2.725 (7.4)	0.028
DBP	69 ± 8.3	83 ± 17.1		−2.271 (7.4)	0.055
HR	86 ± 11.2	85 ± 6.6		0.228 (71)	0.820
D_I_	44.2 ± 17.3	161.8 ± 90.3		−3.674 (7.1)	0.008
D_E_	40.1 ± 16.9	154.5 ± 89.6		−3.607 (7.1)	0.009
Fibers	4.12 ± 1.13	7.22 ± 2.5		−3.530 (7.4)	0.009
SAT kg	3.48 ± 1.11	13.09 ±7.5		−3.643 (7.0)	0.008
SAT %	12.7 ± 3.5	23.68 ± 6.4		−4.737 (7.5)	0.002
*correlations*	SBP	DBP	HR		
D_I_	0.550 **	0.402 **	−0.013		
D_E_	0.548 **	0.400 **	−0.009		
Fibers	0.380 **	0.298 **	−0.129		

Abbreviations: SBP: systolic blood pressure, DBP: diastolic blood pressure, HR: heart rate, D_I_: sum SAT including fibers (mm), D_E_: sum SAT excluding fibers (mm), SAT in kg and %. Note: ** flag significant correlations (*p* < 0.01, respectively).

**Table 4:** Hemodynamic measures and subcutaneous adipose tissue (SAT) in adolescents (13–17 years) with normal weight, overweight or obesity.

BMI Adolescents	<85th pc	≥85th < 95th pc	≥95th	Total	
Girls	36 (54.5%)	19 (28.8%)	11 (16.7%)	66 (100%)	
Boys	14 (70.0%)	2 (10.0%)	4 (20.0%)	20 (100%)	
Total adolescents	50 (51.8%)	21 (24.4%)	15 (17.4%)	86 (100%)	
	mean ± SD	mean ± SD	mean ± SD	F _(df)_	*p*
SBP	113 ± 8.2	124 ± 14.1	126 ± 11.1	14.043	<0.001
DBP	66 ± 6.9	68 ± 11.2	70 ± 8.1	1.533	0.222
HR	80 ± 11.7	77 ± 8.3	80 ± 7.9	0.929	0.399
D_I_	82.3 ± 38.2	171.7 ± 36.8	226.7 ±49.0	91.399	<0.001
D_E_	76.7 ± 37.5	163.6 ± 36.7	217.6 ± 48.0	89.514	<0.001
Fibers	5.5 ± 1.5	8.2 ± 2.9	9.1 ± 4.0	16.348	<0.001
SAT kg	8.02 ± 3.1	15.6 ± 3.0	21.9 ± 5.5	96.301	<0.001
SAT %	15.8 ± 5.1	23.8 ± 3.2	26.1 ± 2.8	45.10	<0.001
*correlations*	SBP	DBP	HR		
D_I_	0.534 **	0.262 *	0.040		
D_E_	0.525 **	0.252 *	0.042		
Fibers	0.528 **	0.371 **	−0.020		

Abbreviations: SBP: systolic blood pressure, DBP: diastolic blood pressure, HR: heart rate, D_I_: sum SAT including fibers (mm), D_E_: sum SAT excluding fibers (mm), SAT in kg and %. Note: *, ** flag significant correlations (*p* < 0.05, *p* < 0.01, respectively).

## Data Availability

All de-identified individual participant data, including the study protocol and informed consent form, collected during the study will be shared on request immediately following publication and until three years following publication. Researchers who provide a methodologically sound proposal may send their request to the corresponding author.

## Supplementary Materials

The following are available online at https://www.mdpi.com/article/10.3390/biology10050449/s1, Table S1: Anthropometry, body indices and SAT in four cases excluded from descriptive statistics; Figure S1: SAT thicknesses (mm) at the eight standardized sites in female (A) and male (B) children and adolescents; Table S2: Correlation of subcutaneous adipose tissue (SAT) and anthropometric measures at eight sites in children (7–10 years) and adolescents (13–17 years); Table S3: Correlation between SAT at the eight standardized sites and hemodynamic parameters; Table S4: Descriptive statistics and correlation between SAT measures based on eight-site, five-site and four-site measurements in the total cohort (*n* = 162).
