# Supplementary material for: Subcutaneous Adipose Tissue Measured by B-Mode Ultrasound to Assess and Monitor Obesity and Cardio–Metabolic Risk in Children and Adolescents
Source: Biology (Basel). 2021 May 20;10(5):449. doi: 10.3390/biology10050449 (PMC8161059; doi:10.3390/biology10050449)
Supplement: Supplementary file 1 [file biology-10-00449-s001.zip › biology-1199381-supplementary.pdf]

Table S1. Anthropometry, body indices and SAT in four cases excluded from descriptive statistics.

	Girl	Boy	Girl	Boy
age	10	11	11	15
Anthropometric measures				
body height (m)	1.31	1.43	1.48	1.66
body weight (kg)	60.69	65.48	86.83	126.20
sitting height (m)	0.65	0.73	0.78	0.88
leg length (m)	0.74	0.81	0.81	0.99
neck circumference (cm)	29	34	32	41
chest circumference (cm)	65	83	95	119
mid upper arm circ. (cm)	34	32	36	40
waist circumference (cm)	91	96	96	123
hip circumference (cm)	106	101	124	132
thigh circumference (cm)	54	56	67	80
calf circumference (cm)	36	36	50	48
ankle circumference (cm)	23	27	32	29
Body indices				
BMI (kg/m^2)	35.64	31.89	39.43	45.80
MI_I (0.53 m/(hs))	38.49	33.18	40.31	45.79
Cormic index	0.50	0.51	0.53	0.53
WHt ratio (m)	0.69	0.57	0.61	0.73
SAT measures (in mm)				
upper abdomen (UA)	42.49	35.89	49.86	51.46
lower abdomen (LA)	46.51	43.81	61.16	73.34
front thigh (FT)	32.20	26.56	42.53	34.25
lateral thigh (LT)	66.48	52.21	50.25	55.55
medial calf (MC)	15.40	19.20	32.44	24.69
erector spinae (ES)	28.15	44.76	29.41	39.57
distal triceps (DT)	25.37	14.05	12.79	22.22
brachioradialis (BR)	13.10	10.64	10.89	14.17
D_I	269.69	247.11	289.33	315.25
D_E	262.73	238.91	280.97	297.56
Fibres	6.96	8.20	8.35	17.69
SAT (kg)	18.51	19.42	24.66	33.28
SAT (%)	30.50	29.66	28.40	26.37
Hemodynamic measures				
SBP	126	149	185	156
DBP	70	91	106	77
HR	85.7	84.7	87.7	84.3

Abbreviations: BMI: body mass index; MI: mass index; WHt: waist to height ratio; SAT: subcutaneous adipose tissue; D_I: sum SAT inkluding fibres; D_E: sum SAT excluding fibres, SAT in kg and in %, SBP: systolic blood pressure, DBP: diastolic blood pressure, HR: heart rate.

Figure S1. SAT thicknesses (mm) at the eight standardized sites in female (**A**) and male (**B**) children and adolescents.

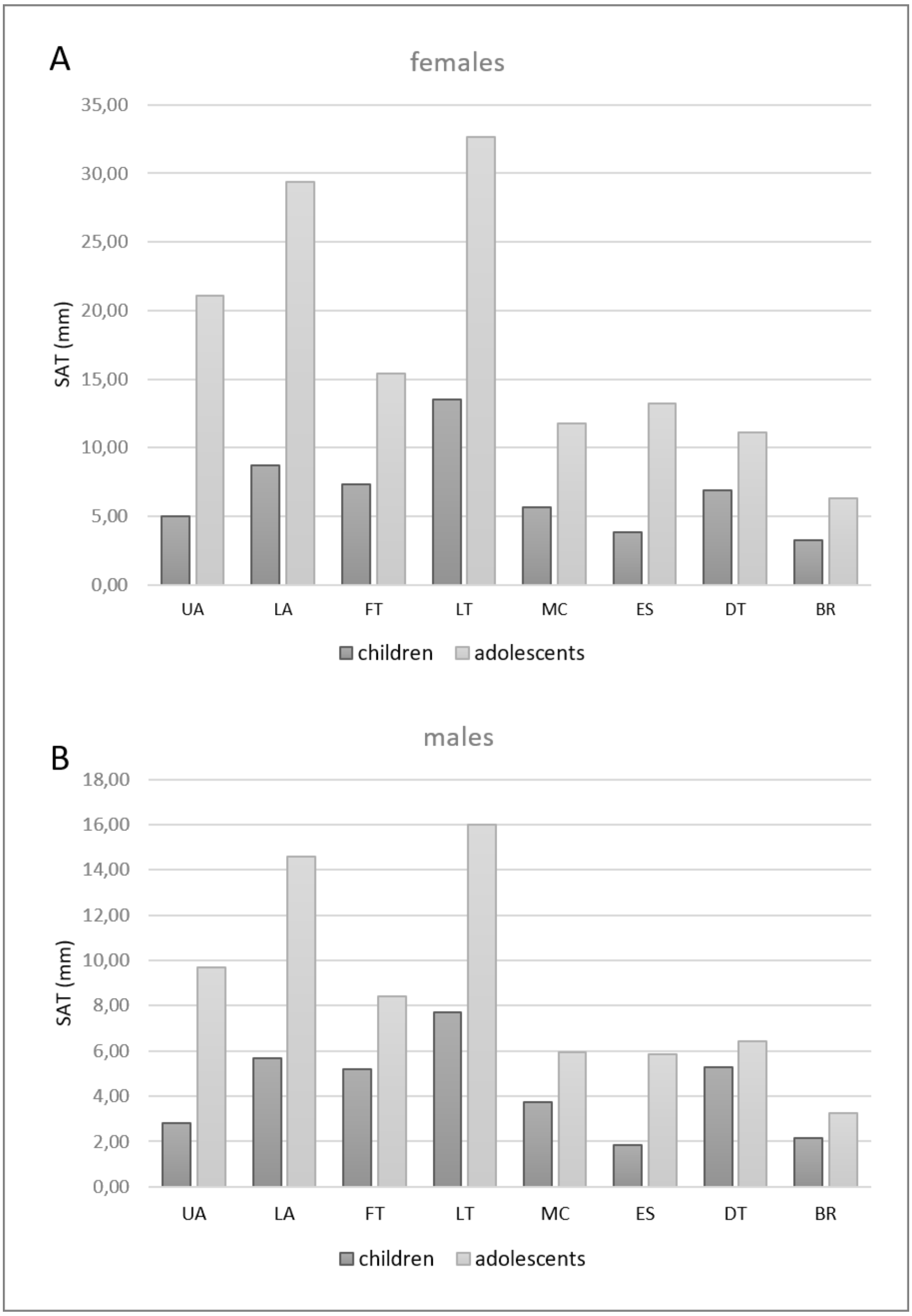

Abbreviations: UA: upper abdomen, LA: lower abdomen, FT: frontal thigh, LT: lateral thigh, MC: medial calf, ES: erector spinae, DT: distal triceps, BR: brachioradialis.

Table S2. Correlation of subcutaneous adipose tissue (SAT) and anthropometric measures at eight sites in children (7-10 years) and adolescents (13–17 years).

**Children**	**Neck**	**Chest**	**MUA**	**Waist**	**Hip**	**Thigh**	**Calf**	**Ankle**	**BMI**
UA	.345**	.473**	.539**	.595**	.531**	.648**	.481**	.192	.687**
LA	.385**	.544**	.608**	.659**	.643**	.702**	.565**	.270*	.757**
FT	.271*	.286*	.477**	.426**	.443**	.620**	.379**	.112	.502**
LT	.243*	.322**	.562**	.441**	.465**	.694**	.473**	.187	.539**
MC	.235*	.308**	.478**	.465**	.497**	.662**	.488**	.154	.505**
ES	.340**	.411**	.535**	.518**	.487**	.618**	.387**	.291*	.539**
DT	.230	.383**	.534**	.457**	.443**	.688**	.482**	.183	.491**
BR	.286*	.352**	.554**	.454**	.478**	.660**	.469**	.118	.651**
D_I	.336**	.449**	.611**	.579**	.572**	.747**	.533**	.225	.670**
D_E	.337**	.448**	.611**	.580**	.580**	.743**	.521**	.217	.666**
Fibres	.169	.254*	.332**	.300**	.192	.470**	.459**	.230	.417**
SAT (kg)	.465**	.583**	.637**	.658**	.680**	.815**	.651**	.373**	.745**
SAT (%)	0.222	.279*	.543**	.371**	.426**	.671**	.445**	0.151	.461**
BMI	.499**	.613**	.498**	.711**	.696**	.680**	.673**	.333**	1
**Adolescents**	**Neck**	**Chest**	**MUA**	**Waist**	**Hip**	**Thigh**	**Calf**	**Ankle**	**BMI**
UA	.440**	.869**	.782**	.851**	.819**	.782**	.718**	.596**	.859**
LA	.494**	.881**	.791**	.887**	.856**	.809**	.741**	.616**	.863**
FT	.389**	.796**	.731**	.739**	.867**	.833**	.794**	.696**	.860**
LT	.326**	.728**	.680**	.706**	.796**	.690**	.609**	.528**	.765**
MC	.335**	.764**	.722**	.702**	.834**	.772**	.791**	.698**	.831**
ES	.316**	.795**	.694**	.737**	.817**	.714**	.634**	.566**	.864**
DT	.373**	.780**	.756**	.726**	.827**	.762**	.710**	.640**	.800**
BR	.300**	.762**	.745**	.729**	.759**	.707**	.684**	.568**	.788**
D_I	.429**	.884**	.808**	.852**	.896**	.828**	.763**	.652**	.906**
D_E	.420**	.881**	.803**	.847**	.895**	.825**	.762**	.650**	.905**
Fibres	.422**	.507**	.518**	.565**	.453**	.481**	.403**	.379**	.486**
SAT (kg)	.486**	.905**	.826**	.874**	.922**	.872**	.825**	.726**	.938**
SAT (%)	.308**	.788**	.717**	.730**	.841**	.714**	.674**	.539**	.779**
BMI	.494**	.886**	.814**	.840**	.906**	.882**	.820**	.746**	1

Abbreviations: MUA: mid upper arm circumference, UA: upper abdomen, LA: lower abdomen, FT: frontal thigh, LT: lateral thigh, MC: medial calf, ES: erector spinae, DT: distal triceps, BR: brachioradialis; D_I: sum SAT including fibres; D_E: sum SAT excluding fibres, SAT (subcutaneous adipose tissue) in kg and in %; BMI: body mass index. *,** correlation is significant at $p < .05$/$p < .01$, respectively.

Table S3. Correlation between SAT at the eight standardized sites and hemodynamic parameters.

**Children**	**SBP**	**DBP**	**HR**
upper abdomen (UA)	.548**	.405**	-.014
lower abdomen (LA)	.590**	.423**	-.029
front thigh (FT)	.554**	.404**	.002
lateral thigh (LT)	.459**	.338**	-.017
medial calf (MC)	.576**	.432**	.005
erector spinae (ES)	.528**	.410**	-.021
distal triceps (DT)	.356**	.200	.039
upper abdomen (UA)	.519**	.380**	-.027
DI	.550**	.402**	-.013
DE	.548**	.400**	-.009
Fibres	.380**	.298**	-.129
SAT (kg)	.620**	.472**	-.021
SAT (%)	.425**	.302*	.033
**adolescents**	SBP	DBP	HR
upper abdomen (UA)	.537**	.258*	-.005
lower abdomen (LA)	.547**	.260*	.037
front thigh (FT)	.408**	.252*	.054
lateral thigh (LT)	.433**	.222*	-.001
medial calf (MC)	.404**	.183	.091
erector spinae (ES)	.514**	.226*	.117
distal triceps (DT)	.490**	.225*	.042
upper abdomen (UA)	.506**	.254*	.047
DI	.534**	.262*	.040
DE	.525**	.252*	.042
Fibres	.528**	.371**	-.020
SAT (kg)	.555**	.264*	.049
SAT (%)	.436**	.205	.064

Abbreviations: SBP: systolic blood pressure (mmHg), DBP: diastolic blood pressure (mmHg), HR: heart rate, SAT: subcutaneous adipose tissue (mm); DI: sum SAT including fibres DE: sum SAT excluding fibres, SAT in kg an in %. Note: *, ** flag significant correlations (p < .05, <.01, respectively).

Table S4. Descriptive statistics and correlation between SAT measures based on 8-site, 5-site and 4-site measurements in the total cohort (n= 162).

	**mean**	**SD**	**min.**	**max.**
D_I 8-sites	95.19	71.04	14.98	315.25
D_E 8-sites	89.49	69.29	11.09	303.11
Fibres 8-sites	5.70	2.64	2.21	17.69
SAT (kg) 8-sites	8.73	6.72	1.84	35.24
SAT (%) 8-sites	16.97	6.41	4.76	31.83
D_I 5-sites	72.80	55.88	11.73	247.26
D_E 5-sites	68.45	54.64	8.11	238.87
Fibres 5-sites	4.35	2.00	1.58	15.20
SAT (kg) 5-sites	8.04	6.18	1.67	31.19
SAT (%) 5-sites	15.73	6.12	4.93	30.28
D_I 4-sites	65.23	51.81	9.25	226.72
D_E 4-sites	61.20	50.46	7.02	218.53
Fibres 4-sites	4.03	2.11	1.37	15.19
SAT (kg) 4-sites	8.15	6.44	1.66	31.25
SAT (%) 4-sites	15.69	6.30	4.67	31.09

	**D_I 8-sites**	**D_E 8-sites**	**Fibres 8-sites**	**SAT (kg) 8-sites**	**SAT (%) 8-sites**
D_I 5-sites	.998**	.997**	.668**	.978**	.939**
D_E 5-sites	.997**	.998**	.648**	.977**	.938**
Fibres 5-sites	.633**	.614**	.964**	.654**	.588**
SAT (kg) 5-sites	.983**	.982**	.689**	.995**	.895**
SAT (%) 5-sites	.944**	.944**	.633**	.894**	.987**
D_I 4-sites	.996**	.995**	.685**	.976**	.932**
D_E 4-sites	.996**	.996**	.663**	.974**	.933**
Fibres 4-sites	.632**	.611**	.958**	.666**	.570**
SAT (kg) 4-sites	.979**	.977**	.716**	.993**	.885**
SAT (%) 4-sites	.945**	.944**	.638**	.892**	.981**

Abbreviations: SAT: subcutaneous adipose tissue; D_I: sum SAT including fibres; D_E: sum SAT excluding fibres, SAT in kg and in %. Note: *, ** flag significant correlations (p < .05, <.01, respectively).
